# Editorial: Verification and Validation of *in silico* Models for Biomedical Implantable Devices

**DOI:** 10.3389/fmedt.2022.856067

**Published:** 2022-03-10

**Authors:** Lorenza Petrini, Giancarlo Pennati, Dimitrios I. Fotiadis

**Affiliations:** ^1^Department of Civil and Environmental Engineering, Politecnico di Milano, Milan, Italy; ^2^Laboratory of Biological Structure Mechanics, Department of Chemistry, Materials and Chemical Engineering “Giulio Natta”, Politecnico di Milano, Milan, Italy; ^3^Unit of Medical Technology and Intelligent Information Systems, Department of Materials Science and Engineering, University of Ioannina, Ioannina, Greece; ^4^Department of Biomedical Research Institute-FORTH, Institute of Molecular Biology and Biotechnology, University of Ioannina, Ioannina, Greece

**Keywords:** V&V40, numerical simulations, *in vitro* experiments, *in vivo* data, medical devices assessment

Nowadays, after the publication by ASME of V&V40 international standard “Assessing Credibility of Computational Modeling Through Verification and Validation: Application to Medical Devices” (2018), when computational models are used to assess medical device performance, the establishment and communication of their risk-informed credibility is expected. In the framework defined by V&V40, the credibility of the *in silico* model is assessed with respect to a specific Context Of Use (COU), which defines the specific role and purpose of the model and how the use of its predictive capabilities can answer a well-defined Question Of Interest (QOI).

A fundamental step in this process is the validation of the model through a comparison of *in silico* predictions with *in vitro* or *in vivo* data. The rigor and agreement of output comparison are combined in a single gradation, based on the risk level that can be reached when incorrect decisions and undesirable results derive from the use of the model. In particular, the accepted mismatch between computational results and experimental data varies from <20%, when the risk of the model is low, to <5%, when the risk of the model is high.

The procedure proposed in V&V40 is undoubtedly an important guideline for biomedical researchers and medical device companies. However, its applicability leaves some questions open, as discussed in the papers collected in this Research Topic and summarized below.

The selection of the comparator is a very critical point, in particular when the device under study significantly interacts with a biological counterpart during its functioning. In the case of cardiovascular implantable devices (stents and valves), experimental tests are commonly performed involving only the device (such as radial compression, three-point bending, and two plate crush tests). These tests, performed to evaluate the representative mechanical properties of the considered device, provide optimal comparators for *in silico* models and, for well-performed simulations, a very high rigor with an error lower than 5% can be obtained, as described in the paper “*Application of in-silico platform for development and optimization of fully bioresorbable vascular scaffold designs*” by Milosevic et al..

If a further step is included, where the experiments consider the implantation of the device in mock-up tissues or organs (e.g., arterial vessels), a more complex situation has to be faced due to the uncertainties introduced in the comparator, as reported in the paper “*How to validate in silico deployment of coronary stents: strategies and limitations in the choice of the comparator*” by Berti et al.. As a testing environment that realistically mimics the *in vivo* conditions is required, complex 3D printed mock-up vessels are needed, which involve a large geometrical and material variability as well as some difficulties in data measurement. This, in turn, makes not trivial the comparison between the *in silico* and the experimentally collected data, as the observed data mismatch derives from both errors of *in silico* predictions and comparator uncertainties.

When patient-specific data acquired *in vivo* are used as a comparator (or even as model inputs), the high level of uncertainties intrinsic to clinical data acquisition strongly affects the numerical results, as demonstrated in the paper “*On the role and effects of uncertainties in cardiovascular in-silico analyses*” by Celi et al.. This suggests that when numerical tools aim to assist practitioners at the preoperative planning stage and during the intervention, their accuracy has to be defined for each case based on compatibility with clinical specifications and expectations, as suggested in the paper “*Evaluation and Verification of Fast Computational Simulations of Stent-Graft Deployment in Endovascular Aneurysmal Repair*” by Pionteck et al..

The comparator choice and use are also strictly related to the QOI. It is not obvious that a model validated with respect to a specific comparator can be used to get information about other conditions. This is the case presented in “*Cerebral Aneurysm Occlusion at 12-Month Follow-Up After Flow-Diverter Treatment: Statistical Modeling for V&V With Real-World Data*” by Narata et al., where a computational model of cerebral aneurysms treatment through Flow-Diverter (FD) is presented. In particular, the capability of the *in silico* model to predict also the aneurysm post-treatment evolution during follow-up is discussed and validated, comparing the device porosity estimated numerically to *in vivo* measurements.

For the more mature models (as the ones based on pharmacological therapies), it is possible to consider their use in supplementing clinical trials. Proper exploitation of *in silico* data could allow the decrease of size and duration of the clinical trials, potentially speeding up the commercialization of new interventions and reducing their costs. Accordingly, another critical aspect that has to be faced in the process of risk-informed credibility assessment of a computational model is how to correctly incorporate relevant information from *in silico* experiments into clinical trials. Indeed, the two datasets have to be combined without overwhelming the information from *in vivo* trials, to improve the precision in the evaluation of the clinical endpoints without biasing the clinical decision. In this direction, Kiagias et al. proposed a suitable methodology in their study “*Bayesian Augmented Clinical Trials in TB Therapeutic Vaccination*.”

In conclusion, in this Research Topic, a number of studies were collected to present some recent analyses about the practical application of V&V40. Globally, these investigations show that despite a detailed procedure being reported in the guidelines, further insights are required to make it applicable to various medical devices and therapies, taking into account the different COUs and QOIs associated with their final *in vivo* use.

## Author Contributions

All authors listed have made a substantial, direct, and intellectual contribution to the work and approved it for publication.

## Funding

This project has received funding from the European Union's Horizon 2020 research and innovation program under grant agreement n° 777119.

## Conflict of Interest

The authors declare that the research was conducted in the absence of any commercial or financial relationships that could be construed as a potential conflict of interest.

## Publisher's Note

All claims expressed in this article are solely those of the authors and do not necessarily represent those of their affiliated organizations, or those of the publisher, the editors and the reviewers. Any product that may be evaluated in this article, or claim that may be made by its manufacturer, is not guaranteed or endorsed by the publisher.

